# Evaluating phenotypic plasticity of reproductive traits among Korean rice cultivars under diverse climatic conditions

**DOI:** 10.3389/fpls.2026.1697493

**Published:** 2026-03-19

**Authors:** Joon Ki Hong, Sheikh Mansoor, Jeongho Baek, Jiseon Song, Song Lim Kim, Jae Il Lyu, Hyun-Sook Lee, Yong Suk Chung, Kyung-Hwan Kim

**Affiliations:** 1 National Institute of Agricultural Sciences, Rural Development Administration, Jeonju, Republic of Korea; 2 Department of Horticulture, Faculty of Agriculture, Recep Tayyip Erdoğan University, Rize, Türkiye; 3 National Institute of Crop Science, Rural Development Administration, Wanju, Republic of Korea; 4 Department of Plant Resources and Environment, Jeju National University, Jeju, Republic of Korea

**Keywords:** climate change, phenotypic plasticity, rice, traits, yield

## Abstract

Developing climate-resilient cultivars is crucial for maintaining global rice production amid increasing climatic uncertainty. Harnessing phenotypic plasticity provides a promising pathway to enhance crop adaptability; however, a comprehensive understanding of how environmental factors shape phenotypic responses remains limited. In this study, we rigorously assessed the phenotypic plasticity of key reproductive traits in 100 Korean rice cultivars representing three distinct maturity groups. We constructed a comprehensive dataset by combining measurements from a precision-controlled plant phenotypic-measuring automated greenhouse (PMAG) with two field environments with contrasting climatic conditions: a dry year (2018) and a wet year (2020). Using *Z*-score standardization and kernel density estimation (KDE), we analyzed trait distributions and their environmental shifts to reveal plasticity patterns across conditions. Panicle length and total seed number exhibited pronounced variability, with medium–late-maturing cultivars generally showing higher mean and median values, suggesting greater potential for enhanced panicle development and yield. Specifically, the mean panicle length increased from a minimum of 17.99 cm in the early-maturing group under 2018 field conditions to a maximum of 22.10 cm in the medium–late group under the 2023 PMAG environment. The number of seeds per panicle also showed an upward trend, reflecting improved reproductive output under the controlled PMAG conditions. Overall, climatic variability, particularly rainfall patterns and controlled environment factors, strongly influenced the manifestation of reproductive traits. This environmental influence was clear in the significant shifts in trait expression observed across the three maturity groups. These results establish a robust multi-environment analytical framework that disentangles environmental–phenotypic relationships in rice. Climatic factors were shown to differentially regulate reproductive development through maturity-dependent plasticity. Early-maturing genotypes exhibited higher environmental sensitivity, whereas medium- and late-maturing groups displayed greater buffering capacity. This provides a predictive basis and a practical breeding strategy for developing climate-resilient rice cultivars suited to future agroecosystems.

## Graphical Abstract

**Figure:**
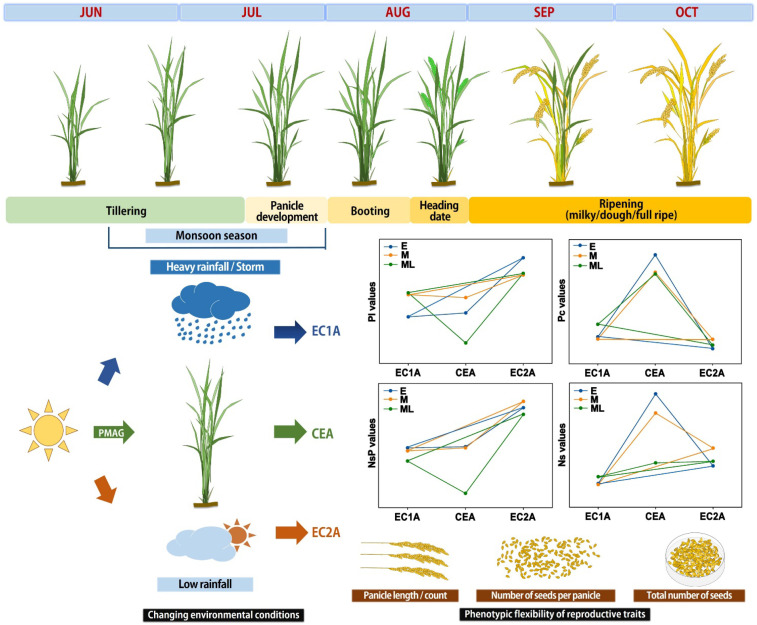
The abstract should ideally be structured according to the IMRaD format (Introduction, Methods, Results and Discussion). Provide a structured abstract if possible. If your article has been copyedited by us, please provide the updated abstract based on this version.

## 1 Introduction

Global climate shifts present unprecedented survival challenges for plant species (Arnold et al., 2019). Under these volatile conditions, individuals capable of manifesting persistent yet reversible adaptations—often referred to as phenotypic plasticity—gain a significant selective advantage. Such organisms can modulate their behavioral, physiological, and morphological traits within a single lifespan, allowing them to navigate environmental fluctuations more effectively than those with rigid phenotypes (Leray et al., 2021; Bashir et al., 2021). Consequently, alterations in plant community structures under shifting environments are driven by both individual phenotypic adjustments and differential survival rates among species (Garnier et al., 2018). These phenotypic variations are fundamentally governed by the intricate interplay between genotype (G), environment (E), and developmental trajectories, as well as the synergistic interactions among these three components (Kusmec et al., 2018).

The more diverse the genotypes studied and the environment measured, the more phenotypic variations can be observed (Kusmec et al., 2018). However, in evaluating the effect of genetic variations in plants on phenotypes in order to cope with environmental changes (Napier et al., 2023), it is difficult to investigate the phenotypic effects of subtle changes in physiological and/or developmental processes due to gene dysfunction, or whether gene function may be obscured by genetic redundancy (Begna, 2021). This is because it reflects the cumulative effect of genetic variations in plants on environmental fitness (i.e., the ability of individuals to survive and reproduce) during plant growth (Wang et al., 2022). In recent years, agriculture has shown that improved single cultivars with uniformity in crop size, shape, and ripeness have been major phenological and management constraints in harvest efficiency and overall crop yield variability caused by environmental changes (González Guzmán et al., 2022; Mansoor et al., 2022; Karunathilake et al., 2023; Sheikh et al., 2024). Choosing crop uniformity carries the risk of single cultivation, which can be attributed to crop loss to environmental changes (Begna, 2021). Generally, plant reaction studies examine the effects of a single environmental factor or treatment but fail to account for the combined effects of multiple complex environmental signals encountered by crops at external sites (Laitinen, 2024).

Rice (*Oryza sativa* L.) is a staple food in East Asia and South Asia as well as across the globe, ranking among the most important products alongside wheat, corn, and barley, and provides a major source of calorie intake for the global population (Alam et al., 2024). Asia is the world’s largest rice-producing continent, accounting for 90.6% of its total production (Bandumula, 2018; FAO, 2021). Rice production in 2021/2022 stood at 515 million tons, with consumption at approximately 519 million tons (USDA Foreign Agricultural Service, 2023). The global population is expected to reach 9 billion by 2050, and rice production must be increased by more than 60% from the present to prepare for food shortages and ensure food security (Daszkiewicz, 2022). Rice has complex agronomic properties that are determined by key components such as the number of panicles per unit area, panicle length, and the number of seeds (Hang et al., 2024). Other properties such as leaf area, height, and width also act as indirect factors affecting grain yield during the cultivation period (Zhao et al., 2022). Currently, rice breeding programs focus on the development of rice varieties with excellent performance, stability, and adaptability under various environmental conditions to develop high-yield varieties under rapidly changing climatic conditions (Li et al., 2019; Wang et al., 2021). In addition, the characteristics of rice varieties were fine-tuned to enhance favorable traits and develop high-yielding varieties (Nakata et al., 2018; Kaewmungkun et al., 2023). Interestingly, the yield of rice varieties depends on their interactions with genotypes and environmental factors (Huang et al., 2021). Most of the rice of commercial varieties in Korea is made by constructing near-isogenic lines (NILs) with artificial crosses between temperate Japonica varieties to develop new varieties, resulting in highly limited genetic variation (Ji et al., 2021; Lee et al., 2023). Grain yield and yield-related characteristics were grown over two planting seasons (early and regularly planted) by dividing three growing regions and Korean rice varieties into three mature groups (Lee et al., 2020, 2023). Due to the narrow genetic variation of commercially grown varieties, the genotypic–environmental interaction effect under relatively similar environmental conditions was not statistically significant (Lee et al., 2020, 2023). However, despite the very narrow genetic diversity, domestic rice varieties showed different levels of phenotypic variation patterns according to genotypic and environmental differences depending on the timing and region of cultivation (Lee et al., 2023). Although most commercial varieties grown in the representative rice cultivation zone in South Korea have minimal interaction effects between narrow genetic variation and environmental invariance, wide phenotypic variation has been observed among varieties grown in various regions (Lee et al., 2023). Therefore, rice varieties under various environmental conditions exhibited phenotypic variations according to genotypes to adapt to the environment (Huang et al., 2021).

Phenotypic plasticity refers to all kinds of environmentally induced phenotypic variation as a general characteristic of all living things and can affect the morphological, physiological, and behavioral aspects of an organism’s phenotype as well as its life and death (Sommer, 2020). Phenotypic plasticity in response to actual environmental signals is a characteristic in itself and depends on the environment, which is controlled by genetic and environmental factors and depends on the species and their traits (Sommer, 2020; Laitinen, 2024). Phenotypic variations can be caused by environmental differences, such as non-uniformity in nutrient concentration or competition in adjacent plants (Zhang et al., 2025), or by differences in gene transcription, expression, and transcription levels resulting from non-deterministic characteristics of molecular dynamics (Suhorukova et al., 2025). Understanding the causes of phenotypic variation is also important in botany, evolutionary biology, the agricultural industry, and other biological fields (Sanderson et al., 2023). Recent evolutionary studies have suggested that phenotypic variability may allow rapid adaptation to new environmental conditions through bet-hedging strategies (Sekajova et al., 2025) and may exhibit improved suitability and adaptability in fluctuating environments (Henry, 2020).

Phenotypic plasticity allows plants to adapt to new conditions through mechanisms such as natural selection and migration, which are not mutually exclusive (Nielsen and Papaj, 2022). For sessile organisms like plants, phenotypic plasticity provides a critical means to rapidly optimize growth and development under fluctuating conditions (Laitinen, 2024). This inherent characteristic, governed by genetic factors and varying by species and trait, enables responses to environmental signals (Pérez-Ramos et al., 2019; Hou et al., 2025). Such plasticity generates the phenotypic variability that underpins the productivity and survival of rice cultivars in the face of climate change. To understand these adaptive responses in rice, this study moves beyond analyzing the impact of a single variable (e.g., water supply). Instead, it conducts a comparative analysis of genetic responses across two fundamentally different environmental paradigms. The first is the “dynamic-stochastic field paradigm,” characterized by unpredictable variability. The second is the “static-optimized CEA paradigm,” which pursues optimization through homeostasis. Through this comparative framework, we aim to elucidate how each environmental paradigm differentially influences the expression of phenotypic plasticity and the realization of genetic potential in rice cultivars.

Despite the known impacts of thermal stress on rice, a comprehensive evaluation of how phenotypic plasticity varies across different maturity groups in Korean germplasm is lacking. We hypothesized that the degree of plastic response in reproductive traits is genotype-dependent and varies significantly between controlled greenhouse environments and fluctuating field conditions.

## 2 Materials and methods

### 2.1 Plant materials and weather information

Based on a previous study (Lee et al., 2020), this study selected 100 cultivars with superior quantitative and stability (30 early-, 30 medium-, and 40 medium–late-maturing group according to their maturing characteristics) from a total of 300 Korean rice cultivars released by the National Institute of Crop Science (NICS), Rural Development Administration (RDA) (Lee et al., 2023). Specific information about the rice cultivars is shown in **Supplementary Table S1**.

To assess the impact of climatic variability on rice reproductive traits across disparate growing seasons, field experiments were conducted from May (sowing) to October (harvest) in 2018 and 2020 at the NICS experimental stations in Jeonju (35°84′N, 127°05′E). For these field trials, each cultivar was established in three independent biological replicates, with each replicate assigned to a distinct growing compartment (plot) serving as the experimental unit. In parallel, the 2023 controlled environment study at the PMAG (National Institute of Agricultural Sciences, Jeonju) was performed using three independent biological replications (sets), wherein individual pots functioned as the experimental units to ensure statistical independence and mitigate positional effects within the automated system (Hong et al., 2025).

The seeds were germinated at 25 °C for 3 days following a 10-min hot water treatment at 60 °C. Subsequently, the germinated seeds were sown in 50-hole seedling trays (overall dimensions: 54 cm × 28 cm; individual cells: truncated square pyramid shape, top edge 5 cm, base edge 3 cm, height 5 cm, volume 81.7 cm³). Seedlings were then transplanted into GS190 pots, which are characterized by a truncated conical shape (upper diameter 19 cm, lower diameter 15 cm, height 15 cm, total volume 3,420 cm³). The plants were cultivated under a 14-h light/10-h dark photoperiod at 32 °C/22°C, with a light intensity of 450 µmol/m²/s and 52% relative humidity (**Supplementary Figure S1**). For the cultivation substrate, a rice-specific commercial substrate (Chalgama; Cham-gru Haesa, Hongseong, Republic of Korea), specifically formulated for rice cultivation, was utilized. The substrate was precisely composed of the following components: cocopeat (17.748%), vermiculite (37%), perlite (13%), peat moss (11%), decomposed granite (9%), zeolite (6%), diatomite (6%), quaternary compound fertilizer (0.22%), sulfur powder (0.03%), wood charcoal (0.001%), and a wetting agent (0.001%). To ensure experimental precision and avoid intra-specific nutrient competition, a single 21-day-old seedling was transplanted into each GS190 pot. Throughout the duration of the experiment, the total weight of each pot was meticulously adjusted to 6,700 g via daily irrigation. This gravimetric approach was implemented to standardize the substrate bulk density and maintain the soil moisture at field capacity. By ensuring a consistent volumetric water content across all experimental units, we effectively eliminated water-deficit stress as a confounding factor, thereby confirming that the recorded phenotypic variations in reproductive traits were strictly attributable to the prescribed temperature treatments. Water management, fertilizer spraying, and disease and pest control were carried out according to the standard rice cultivation methods of NICS, RDA (Kim et al., 2016; Lee et al., 2023). It is essential to note that this study was designed as a comparative analysis to investigate the performance of rice under two distinct agricultural paradigms: the stochastic, variable conditions of an open field environment versus the homeostatic, managed system of controlled environment agriculture (CEA). Consequently, the field data from 2018 and 2020 do not serve as a traditional control group for the 2023 CEA experiment. Rather, each environment (field and CEA) represents a distinct experimental condition, and the objective of this study is to compare the resulting ecophysiological responses. The field data provide a baseline for the expression of adaptive plasticity under natural selection pressures, while the CEA data function as the key comparator for exploring how artificial homeostatic conditions recalibrate the physiological responses of the plants.

For the weather information of Jeonju in 2018, 2020, and 2023, daily climate data were obtained and utilized during the rice cultivation seasons (1 May to 31 October) from the Korea Meteorological Administration (https://www.weather.go.kr/neng/index.do). Information on average daily temperature (*T*_avg_), daily maximum temperature (*T*_max_), daily minimum temperature (*T*_min_), relative humidity (RH), hours of daylight (HDL), amount of precipitation (apptn), and number of days with precipitation (npptn) was extracted. The average daily temperature (*T*_avg_) was calculated as *T*_avg_ = (*T*_min_ + *T*_max_)/2. In addition, climate changes were compared and analyzed by measuring average temperature (TG_avg_), maximum temperature (TG_max_), minimum temperature (TG_min_), and relative humidity (RHG) data measured in PMAG. The number of precipitation days was calculated based on the number of days when the daily precipitation was 0.1 mL or more.

### 2.2 Phenotype measurement and data collection

Seed production-related reproductive traits were measured and collected from rice plants in each plot. To evaluate the measurement accuracy, the four phenotypic traits were measured destructively after harvest. Panicle length (Pl) was determined by measuring the length from the panicle node to the end of the main panicle. Panicle count (Pc) was measured by counting the total number of panicles. The number of seeds per panicle count (NsP) was measured by dividing the total number of seeds (Ns) by the total number of panicles. The average values measured at least three times were averaged and displayed (**Supplementary Table S1**).

### 2.3 Phenotypic variation and distance pattern analyses

The values of rice reproductive trait measurement variables were standardized by *Z*-score to evaluate the positions and distances at which cultivars within the three maturing groups moved from the mean according to the cultivation year. Each point in the graph represents the location of the *Z*-score value, where a positive *Z*-score value (+) is on the right side of the mean and a negative value (−) is on the left side of the mean. The resulting values for the lowest and highest *Z*-score ranges of rice varieties represent the phenotypic range of variation.


Z−score value of 18−Pf = A,Z−score value of 20−Pf = B,Z−score value of 23−PMAG = C 

Total magnitude (distance) = |(A−B)|+|(A−C)|+|(B−C)|

### 2.4 Kernel density estimation

To estimate the probability density function of four reproductive growth variables based on kernels, phenotypic variation inference for the population was performed based on reproductive trait data using non-parametric methods. Using separate kernel density estimation (KDE) by applying kernel smoothing (Hildebrandt, 2023; Paçal et al., 2023), probability densities for reproductive traits in cultivation years and cultivated Pf and PMAG were generated for each maturing group. The optimal rate of bandwidth for the distribution series was set to start/end with the binning method set to start/end, and the number of histogram bins was manually set to 60.

To establish a rigorous empirical basis for characterizing phenotypic plasticity, we employed KDE to delineate phenotypic variations; this approach provides a robust statistical framework for identifying regions of maximum data density and enables the precise interpretation of trait distributions across diverse climatic conditions (de Villemereuil et al., 2020). The width, measured at half the peak’s height (full width at half maximum), quantitatively indicates the spread of the distribution, reflecting how widely the data are dispersed. This provides an intuitive measure of the distribution’s width, regardless of its shape (including non-normality). The wide range (*W*) represents the support interval of the density estimate, encompassing the entire range influenced by all kernels.


$$W=(\max(\chi_{i})-\min(\chi_{i}))+2c\;x\;h$$

In the relevant equation, *h* denotes the bandwidth (kernel width), $\chi_{i}$ represents the data points, and *c* is a constant that determines the effective range of the kernel. Several measures describe the distribution’s central tendency.

The mean represents the numerical center or balance point of the entire distribution. The mode corresponds to the peak of the density curve, indicating the most densely populated values and helping to identify clusters or subpopulations within the data. The median is the point where the cumulative probability reaches 50%; because it is insensitive to outliers, it provides a robust measure of central tendency, particularly in asymmetric distributions.

### 2.5 Statistical analysis

Statistical analysis and data visualization of traits associated with seeds were performed using IBM SPSS Statistics version 27 (IBM Corp., Armonk, NY, USA), JAMOVI version 2.5.6 (https://jasp-stats.org, accessed 1 September 2024), JASP version 0.19.1 (https://jasp-stats.org, accessed 15 September 2024), R version 4.3.2 (https://www.r-project.org, accessed 1 September 2024), SAS version 9.4 (SAS Institute Inc, Cary, NC, USA), and SigmaPlot 12.5 (Systat software, GmbH, Germany). Tukey’s *post-hoc* test and one-way ANOVA were used to compare the means. All the analyses were carried out in three replicates. The differences were considered significant when *p ≤*0.05. Using the radar chart from Microsoft Office Excel 2016, we performed an analysis that simultaneously compares the reproductive quantitative variables of rice cultivars consisting of three maturing groups with a baseline metric or common denominator.

To quantify the linear relationship between meteorological factors and reproductive traits, Pearson’s correlation coefficients (*r*) were calculated using R version 4.3.2. A correlation heatmap was generated to visualize the environmental–phenotypic covariance across the three maturity groups, with statistical significance determined at *p <*0.05.

## 3 Results

### 3.1 Response of reproductive growth traits

Rice cultivated under controlled environmental conditions can be used as a great material for statistically analyzing the effect of the surrounding environment on crop growth, thus understanding the effect of genotype–environment interactions. Reproductive traits of 100 Korean rice cultivars, consisting of three maturing groups, were obtained by collecting data from plants grown in PMAG under constant environmental control, including light, temperature, and water supply. Additionally, data were obtained from general field cultivation in rice plots in 2018 and 2020 (**Supplementary Table S1**). In the analyzed reproductive traits, four traits, excluding heading date, were used to analyze the descriptive statistics for three maturing groups (**Supplementary Table S2**). The heading dates of early-, medium-, and medium–late-maturing groups were measured at an average of 83, 94, and 109 days in 18-Pf and similarly measured at 87, 98, and 108 days in 20-Pf, respectively. However, in the 23-PMAG, the heading dates were 71, 85, and 95 days, respectively, earlier than in other cultivation years (**Supplementary Figure S2A**). To reach the simplest model, the most correlated variable, the heading date (Hd, Spearman correlation coefficients > 0.8), used for the classification of maturing groups, was not used for analysis. When excluding one of the correlated variables, we preferentially retained variables that are known to be more important for plant species distributions. In addition, radar chart analysis related to reproductive growth by cultivation year in the three maturing groups showed that there was no difference in the order of heading date, although there was a difference in the timing of heading date depending on the cultivation year (**Supplementary Figure S2B**). While the absolute timing of heading showed significant plasticity, the relative phenological order of the maturity groups (early < medium < late) was robustly maintained across all three environments. These results suggest the presence of a strong main effect of genotype (G), which stably determines the fundamental maturity hierarchy, and a powerful main effect of environment (E), which uniformly accelerated the development of all genotypes. Although our experimental design does not allow for a quantitative partitioning of the variance contribution from the genotype-by-environment interaction (G × E), the observed phenotypes appear to be predominantly determined by the strong main effects of G and E (Laitinen, 2024).

**Figure 1:**
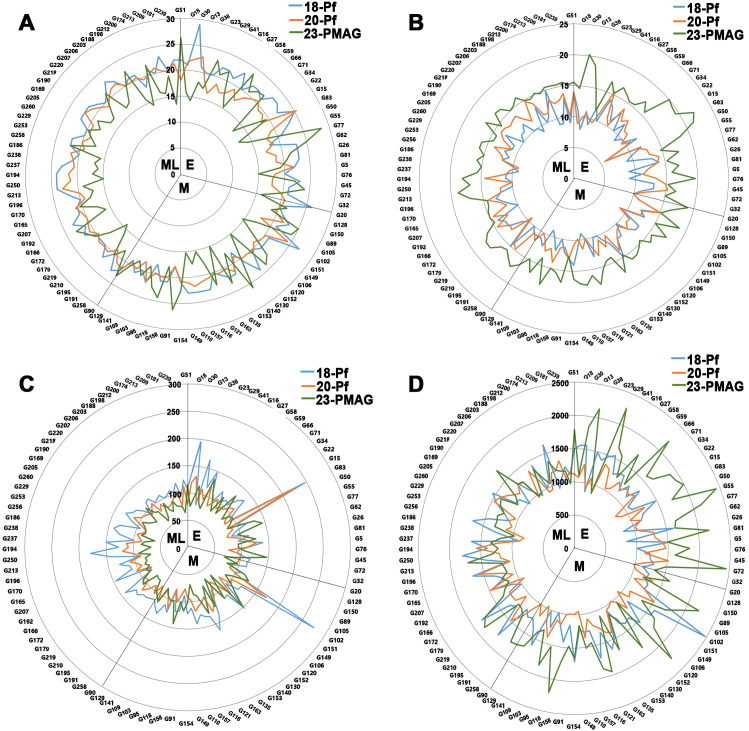
Reproductive trait distribution of 100 Korean rice cultivars (genotypes) consisting of three maturing groups with different cultivation years. Radar charts of the panicle length **(A)**, panicle count **(B)**, number of seeds per panicle **(C)**, and total number of seeds **(D)** in the three maturing groups with different cultivation years. E, early-maturing group; M, medium-maturing group; ML, medium–late-maturing group; 18-Pf, paddy field in 2018; 20-Pf, paddy field in 2020; 23-PMAG, plant phenotypic-measuring automated greenhouse in 2023.

**Figure 2:**
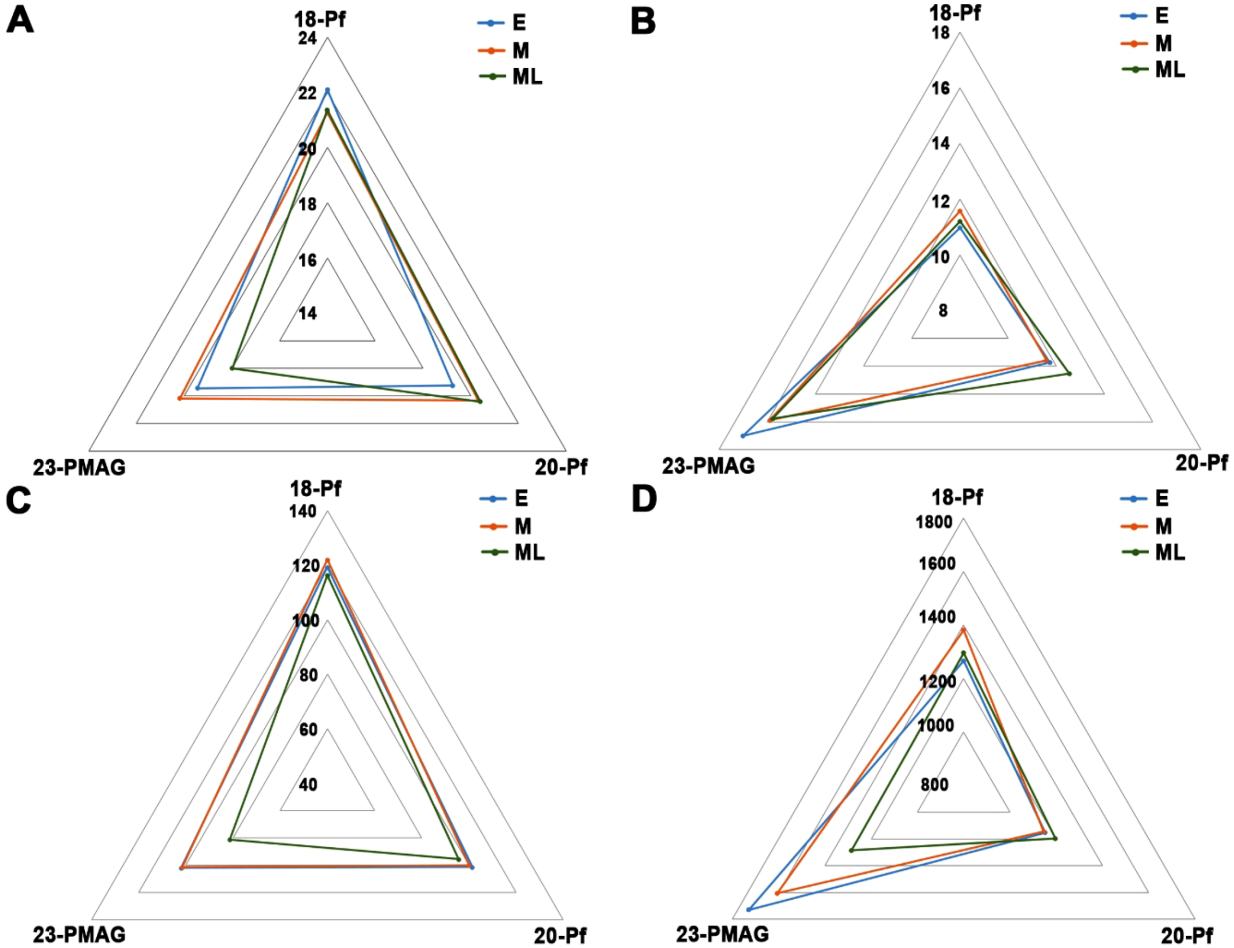
Relationship between the three maturing groups and cultivation years in reproductive traits. Radar chart of the average panicle length **(A)**, average panicle count **(B)**, average number of seeds per panicle **(C)**, and average total number of seeds **(D)** in the three maturing groups with different cultivation years. E, early-maturing group; M, medium-maturing group; ML, medium–late-maturing group; 18-Pf, paddy field in 2018, 20-Pf, paddy field in 2020; 23-PMAG, plant phenotypic-measuring automated greenhouse in 2023.

**Figure 3:**
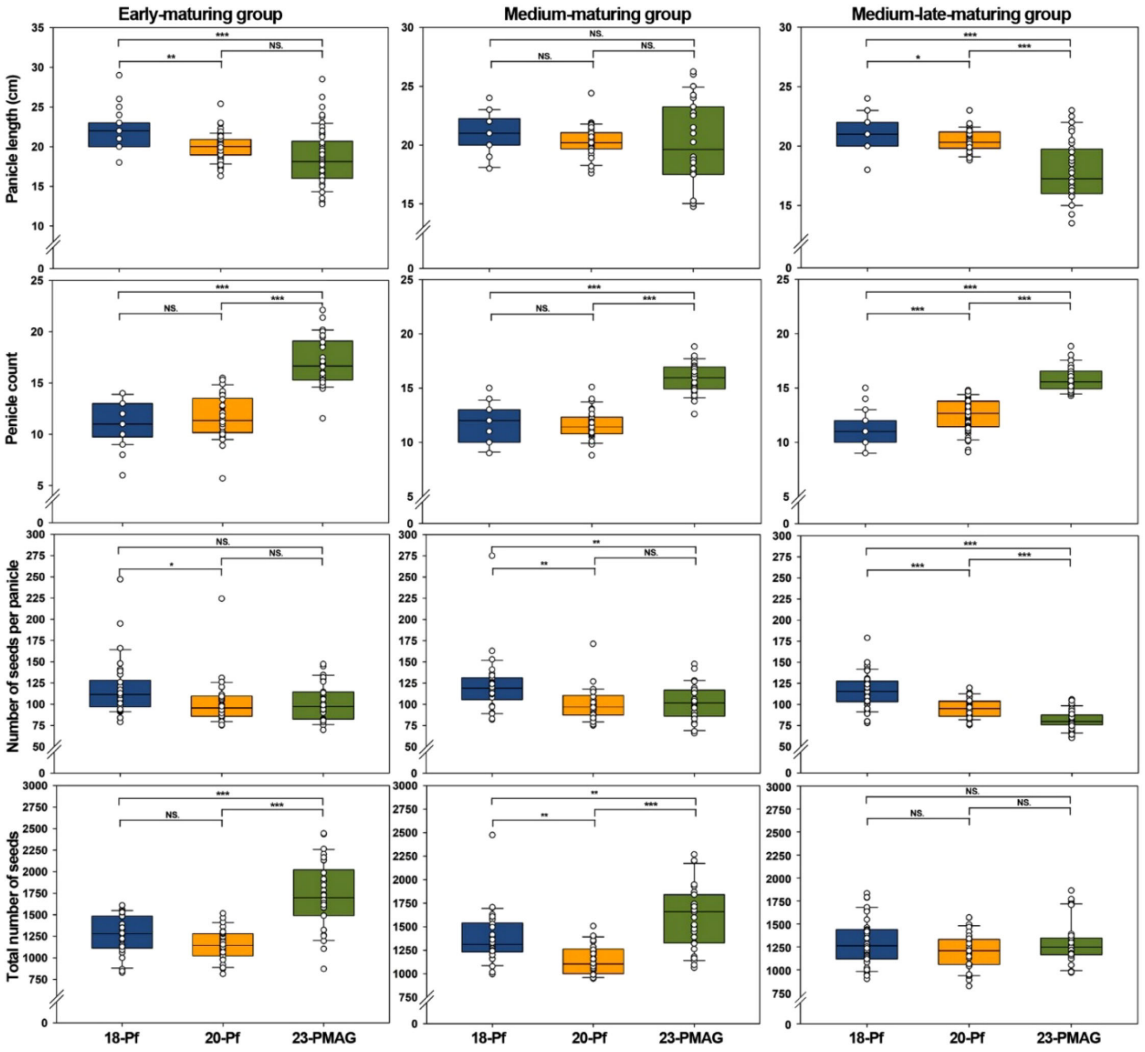
Boxplots displaying the distribution of reproductive traits in the three maturing groups with different cultivation years. The 10th, 50th (median), and 90th quantiles, as well as the minimum and maximum, are shown. E, early-maturing group; M, medium-maturing group; ML, medium–late-maturing group; 18-Pf, paddy field in 2018; 20-Pf, paddy field in 2020; 23-PMAG, plant phenotypic-measuring automated greenhouse in 2023. Significance of the comparisons: NS, no significant; *p < 0.05, **p < 0.01, ***p < 0.001.

To observe the effects of reproductive traits on the Korean rice cultivars across the three maturing groups with cultivation years, a radar chart (**Figures 1**, **2**), histogram (**Supplementary Figure S3**), and boxplot (**Figure 3**) were constructed. The Pl varied according to the cultivation year and showed a low value in 2023-PMAG, and the controlled greenhouse environment showed a significant decrease in Pl (**Figures 1A**, **2B**; **Supplementary Figure S3A**).

On the other hand, the Pc showed a significantly higher growth rate in 23-PMAG compared to 18-Pf and 20-Pf, and the controlled environment changed significantly in Pc, but there was no effect with the cultivation year of the Pf (**Figures 1B**, **2B**; **Supplementary Figure S3B**). In NsP, three maturing groups decreased in 23-PMAG, with the lowest values observed in the medium–late-maturing group. The results showed that the controlled environment showed a positive correlation with Pc and a negative correlation with NsP (**Figures 1C**, **2C**; **Supplementary Figure S3C**). In Ns, 2023-PMAG was found to increase compared to the two cultivation years in the early- and medium-maturing groups, but there was no change in the medium–late-maturing group (**Figures 1D**, **2D**; **Supplementary Figure S3D**). Although the NsP decreased in 2023-PMAG, the Ns of early- and medium-maturing groups appeared to have increased due to a large increase in Pc.

One-way ANOVA (**Supplementary Table S3**) and Tukey’s *post-hoc* test (**Supplementary Table S4**) revealed statistically significant differences (*p* < 0.05) in Pl and Pc in 20-Pf and in all four reproductive traits in 23-PMAG (**Figure 3**).

**Figure 4:**
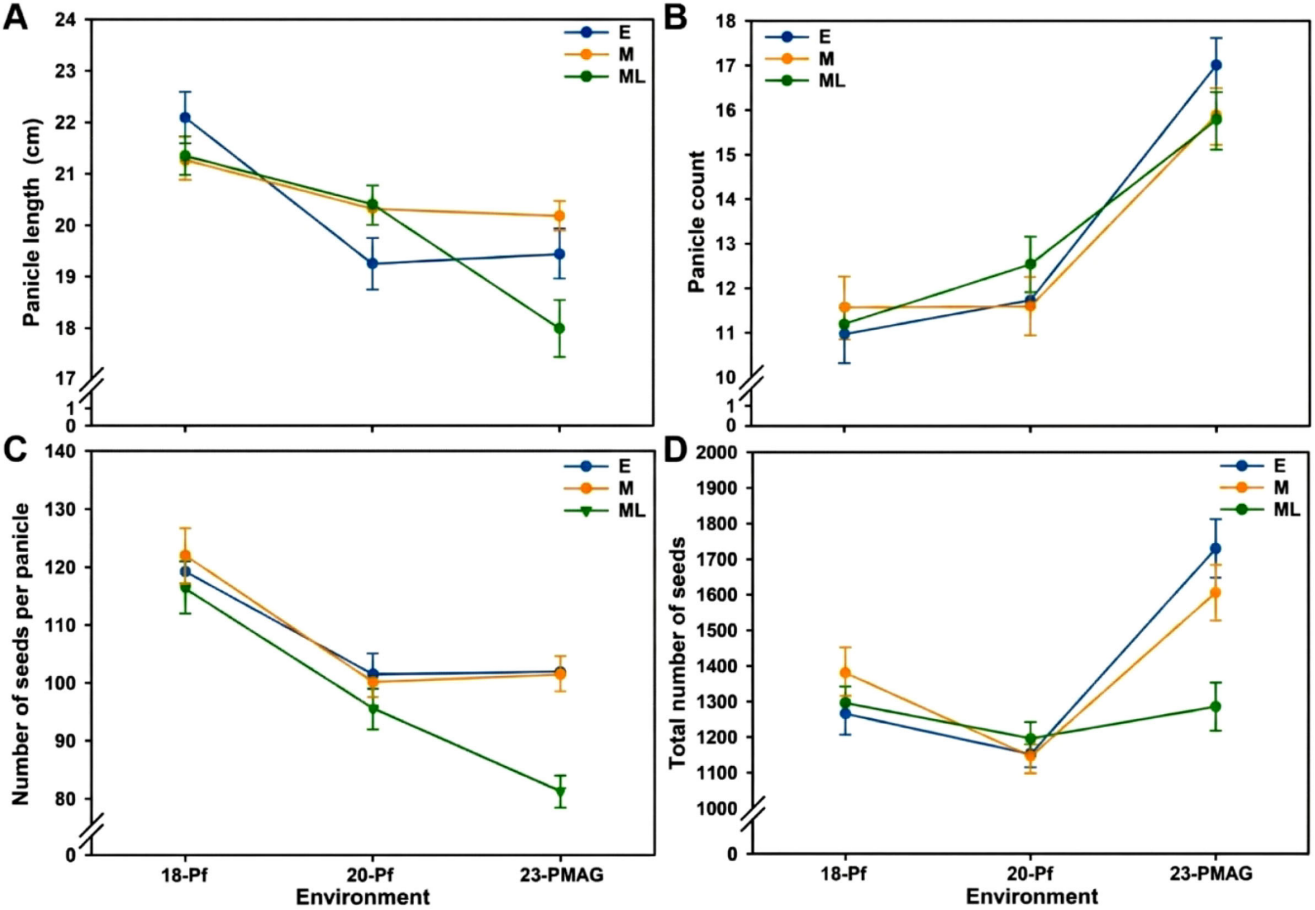
Phenotypic variability in reproductive traits of the three maturing groups. Panicle length **(A)**, panicle count **(B)**, number of seeds per panicle **(C)**, and total number of seeds **(D)** in the three maturing groups with different cultivation years. E, early-maturing group; M, medium-maturing group; ML, medium–late-maturing group; 18-Pf, paddy field in 2018; 20-Pf, paddy field in 2020; 23-PMAG, plant phenotypic-measuring automated greenhouse in 2023. The vertical error bars at each data point represent 95% statistical confidence intervals.

To ascertain the statistical significance of the plastic responses across disparate environments, **Figure 4** was updated to include 95% confidence intervals (CIs). The inclusion of CIs reveals a clear divergence in reproductive strategies among the three maturity groups, particularly when transitioning from the dynamic field environment to the homeostatic CEA system. While the groups displayed overlapping CIs under certain field conditions, the 23-PMAG environment induced a statistically significant differentiation. For instance, the ML group exhibited a pronounced reduction in Pl and NsP that was significantly distinct from the E and M groups, as evidenced by the non-overlapping confidence intervals. This statistically rigorous visualization confirms that the observed phenotypic variations are a direct manifestation of genotype-by-environment (G × E) interactions rather than stochastic variability.

### 3.2 Phenotypic variation position and ranges

In order to determine the variability of genotype phenotypes in the three maturing groups, four reproductive trait values were standardized with a *Z*-score, the left and right positions were checked based on the mean, and the length was analyzed by connecting each position with a line and compared in the lollipop chart and hybrid boxplot.

**Figure 5:**
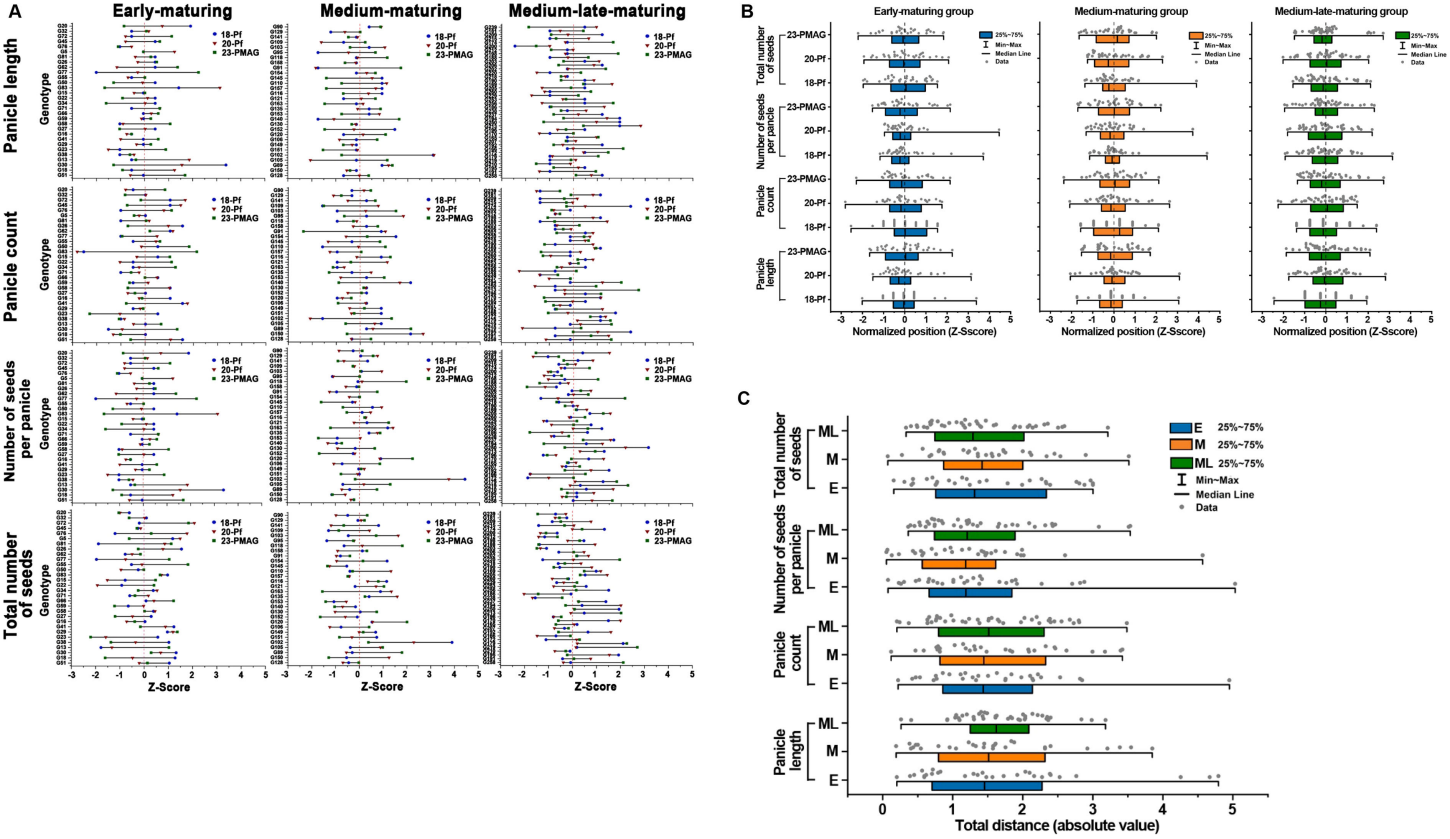
**(A)** Phenotypic variation of reproductive traits according to genotypes within the maturing group. Each lollipop plot shows the positions converted to a standardized *Z*-score for genotypes in the three maturing groups. The color dots represent the results of which direction they moved from the mean at 18-Pf, 20-Pf, and 23-PMAG, and the line represents the distance traveled by connecting the color points. 18-Pf, paddy field in 2018; 20-Pf, paddy field in 2020; 23-PMAG, plant phenotypic-measuring automated greenhouse in 2023. **(B)** Distribution of genotype Z-scores for reproductive traits in the three maturing groups. Genotype distributions in the three maturing groups for reproductive traits under 18-Pf, 20-Pf, and 23-PMAG growing conditions were plotted as hybrid boxplots (boxplots and jitter plots). These plots summarize the distribution of reproductive traits assessed in genotypes of the three maturing groups. 18-Pf, paddy field in 2018; 20-Pf, paddy field in 2020; 23-PMAG, plant phenotypic-measuring automated greenhouse in 2023. **(C)** Total distances of variation of genotype Z-scores for reproductive traits in the three maturing groups. The total distances for the three maturing groups for reproductive traits were plotted as a boxplot and a jitter plot. These plots summarize the total distances of genotypes between the maturing groups of reproductive traits. E, early-maturing group; M, medium-maturing group; ML, medium–late-maturing group.

The position for the genotype Z-score of the three maturing groups showed that they moved from right to left, or from left to right, or only in one direction of left or right, depending on the cultivation year (**Figure 5A**, **Supplementary Table S5**). In addition, the length was measured by connecting each position of the genotype Z-score and represented by a line: the shortest line was 0.053, the longest line was 5.03, and various lengths were found. From the results of this genotype Z-score movement, it is possible to conclude that the cultivars of the three maturing groups react differently depending on the cultivation environment and exhibit phenotypic variability.

In the four reproductive traits, the genotype position of the three maturing groups for the cultivation environment and the complexity of the variability analysis were simply expressed by representing the distribution of genotypes according to the cultivation year (**Figure 5B**). In Pl, the 25% to 75% distribution range according to the cultivation environment was found to be similar in the medium–late-maturing group, but in the early- and medium-maturing groups, it was found to be wide in 23-PMAG. The 25% to 75% distribution in Pc also showed similar ranges in the early-maturing group, while there were different ranges of variation in the medium- and medium–late-maturing groups. In the distribution in NsP, the 25% to 75% area was found to be wide in the early- and medium-maturing groups to 23-PMAG, and in the medium–late-maturing group, 20-Pf was the most widely distributed. The 25% to 75% distribution ranges in Ns also showed similar patterns in the early-maturing group, while the medium-maturing group in 18-Pf and the medium-late-maturing group in 23-PMAG exhibited distinctively narrow ranges.

Next, the total distance of genotypic variability according to the cultivation year in the four reproductive traits was summarized by reconstructing the distribution according to the three maturing groups (**Figure 5C**; **Supplementary Table S5**). In the early-maturing group, the longest ranges were shown in Pl, Pc, and NsP, and the shortest was in Ns. In addition, the distribution of 25% to 75% showed a large range in Pl and Ns. The medium-maturing group showed the shortest length in Pl and a narrow range at 25% to 75% distribution. In NsP, the distribution was 25% to 75%, with the exception of the extreme range of genotypes, and all three groups showed similar ranges. These results suggest that the rice varieties in the three maturing groups exhibit strong genotype–environmental interaction depending on the cultivation environment.

### 3.3 Phenotypic plasticity to cultivation environmental changes

**Figure 6:**
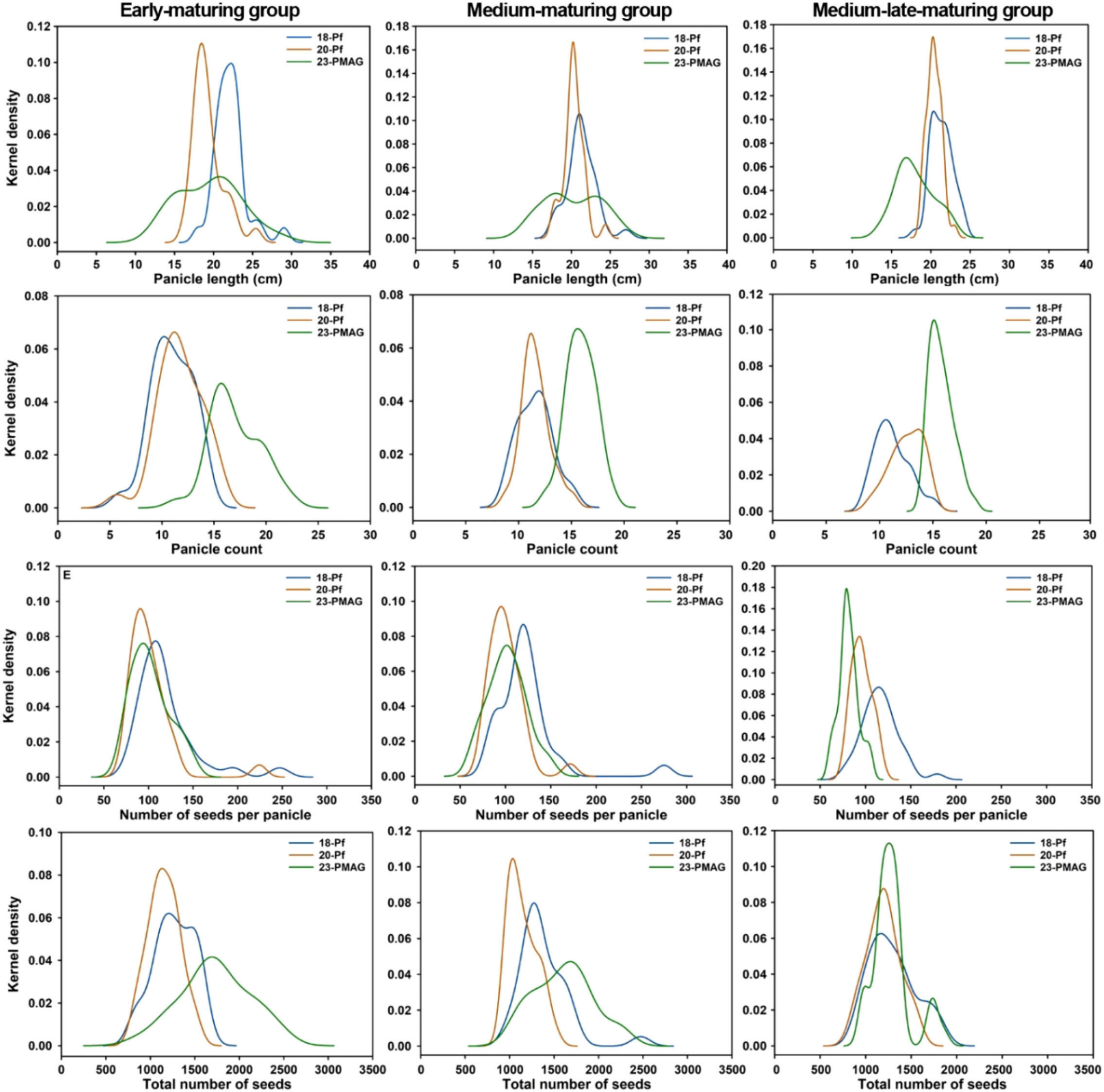
KDE curves with variable optimized bandwidth plotted using histogram stats to visualize peak property distribution for the three maturing groups from 18-Pf, 20-Pf, and 23-PMAG cultivation conditions. 18-Pf, paddy field in 2018; 20-Pf, paddy field in 2020; 23-PMAG, plant phenotypic-measuring automated greenhouse in 2023.

KDE was performed to compare the variability of rice reproductive traits according to the rice Pf, greenhouse, and cultivation year for each maturing group. The diagrams and summary statistics of KDE for confirming each result are shown in **Figure 6**, **Table 1**, respectively. In the KDE curves, reproductive traits by the rice maturing groups showed non-overlapping, left and right movements in the cultivation year and cultivation type or showed changes in height, width, and bimodal. These conditions were found to have a significant effect on the variation in reproductive growth characteristics.

**Table 1:** Summary statistics of KDEs for reproductive traits in 18-Pf, 20-Pf, and 23-PMAG.

Trait	Subscale (unit)	Early-maturing group			Medium-maturing group			Medium–late-maturing group		
		18-Pf	20-Pf	23-PMAG	18-Pf	20-Pf	23-PMAG	18-Pf	20-Pf	23-PMAG
Panicle length	Peak height (probability density)	0.10	0.11	0.037	0.11	0.17	0.040	0.11	0.17	0.070
	Width (cm)	3.78	2.94	11.41	3.20	1.99	9.31	3.52	2.62	5.50
	Wide length (cm)	15.76	14.10	28.62	14.36	10.08	22.81	10.12	6.97	16.74
	Mode (cm)	22.21	18.48	20.74	21.04	20.21	18.01	20.37	20.27	16.88
	Mean (cm)	22.10	19.25	19.44	21.27	20.32	20.18	21.35	20.41	17.99
	Median (cm)	22.00	18.70	19.63	21.00	20.20	19.63	21.00	20.35	17.50
Panicle count	Peak height (probability density)	0.07	0.04	0.05	0.07	0.07	0.05	0.05	0.05	0.11
	Width (count)	5.82	5.49	5.74	4.47	2.57	3.77	3.93	4.22	2.19
	Wide length (count)	14.24	16.65	18.17	11.21	9.99	10.65	10.50	10.30	7.94
	Mode (count)	10.22	11.91	10.61	11.19	11.17	13.64	10.61	13.64	15.10
	Mean (count)	10.97	11.73	15.78	11.57	11.59	15.89	11.20	12.54	15.78
	Median (count)	11.00	11.35	16.66	12.00	11.40	15.96	11.00	12.65	15.56
Number of seeds per panicle	Peak height (probability density)	0.08	0.10	0.08	0.09	0.10	0.08	0.09	0.13	0.18
	Width (count)	47.37	39.02	48.65	35.56	39.26	47.65	43.39	32.29	17.85
	Wide length (count)	242.41	205.76	144.87	254.92	151.11	148.20	156.08	77.19	72.01
	Mode (count)	108.08	90.98	93.79	119.75	95.40	101.53	114.48	93.17	78.94
	Mean (count)	119.23	101.48	101.97	122.03	100.14	101.47	116.28	95.62	81.33
	Median (count)	111.50	95.66	97.30	119.00	96.68	101.74	115.50	95.16	79.83
Total number of seeds	Peak height (probability density)	0.06	0.08	0.04	0.08	0.11	0.05	0.06	0.09	0.11
	Width (count)	630.80	477.58	951.63	463.72	386.91	869.13	580.37	424.61	278.09
	Wide length (count)	1,493.90	1,265.79	2,817.40	2,215.40	1,062.43	2,258.30	1,655.40	1,305.30	1,308.38
	Mode (count)	1,207.32	1,130.12	1,691.02	1,276.84	1,036.96	1,684.14	1,166.04	1,196.24	1,257.44
	Mean (count)	1,265.90	1,152.02	1,729.84	1,380.80	1,145.71	1,606.53	1,295.60	1,196.38	1,285.45
	Median (count)	1,279.00	1,142.66	1,699.25	1,313.00	1,103.90	1,659.50	1,262.00	1,205.40	1,247.00

*18-Pf, paddy field in 2018; 20-Pf, paddy field in 2020; 23-PMAG, plant phenotypic measuring automated greenhouse in 2023.

In the early-maturing group, the KDE of 23-PMAG showed a lower peak width and a wider curve width than the 18-Pf and 20-Pf in Pl, Pc, and Ns traits, except for the NsP trait. In addition, it was confirmed that the curve shifted to the left or right depending on the traits by cultivation conditions, and bimodal distributions appeared. However, NsP showed that the curves overlap under cultivation conditions and that the difference in peak size was small. This is predicted to show a more significant difference in Pl, Pc, and Ns for phenotypic variability with cultivation conditions in the early-maturing group. The peaks in the medium-maturing group are also distinguished by the cultivation year and cultivation type. In Pl and NS traits, the peak height of the 23-PMAG curve was lowered, the width was widened, and the bimodal distribution appeared. On the other hand, in the Pc trait, only the curve moved to the right without a significant difference in width, and it showed a weak bimodal distribution at 18-Pf. Similar to the KDE results of the early-maturing group, NsP had overlapping curves and a small difference in peak size under cultivation conditions. The traits of the medium-maturing group are affected by changes in cultivation conditions, and these have been shown to be the result of variability and movement of curves. In the medium–late-maturing group, the traits show different distributions from the early- and medium-maturing groups depending on the cultivation year and cultivation type. In the Pl trait, the 23-PMAG peak has a low altitude and wide width, but in the Pc and NsP traits, it shows the highest peak and the narrowest width, as well as traits moving from left to right. In the Ns, the difference in width was not significant, but the difference in altitude was shown, and the characteristics were different from those of the previous two groups. Interestingly, it also shows opposite characteristics: the width decreases as the altitude increases. Visual assessment of the response curves for the year and type of cultivation in the three maturing groups revealed that the reproductive characteristic-based response curves showed specificity in altitude, width, position, and morphology for each group at 18-Pf, 20-Pf, and 23-PMAG. These results support the physiological understanding that cultivation environments are strongly related to variability in expression.

### 3.4 Climatic distribution patterns

To understand the association of climate changes to different rice maturing groups’ responses by the cultivation year, we compared and analyzed the weather information of the Meteorological Administration for 2018, 2020, and 2023 and the actual greenhouse environment information data of 2023-PMAG (**Supplementary Figure S4**, **Supplementary Table S6**). How changes in environmental variables in response to climate change would affect crops was first analyzed for changes in daily climate conditions during crop cultivation.

**Figure 7:**
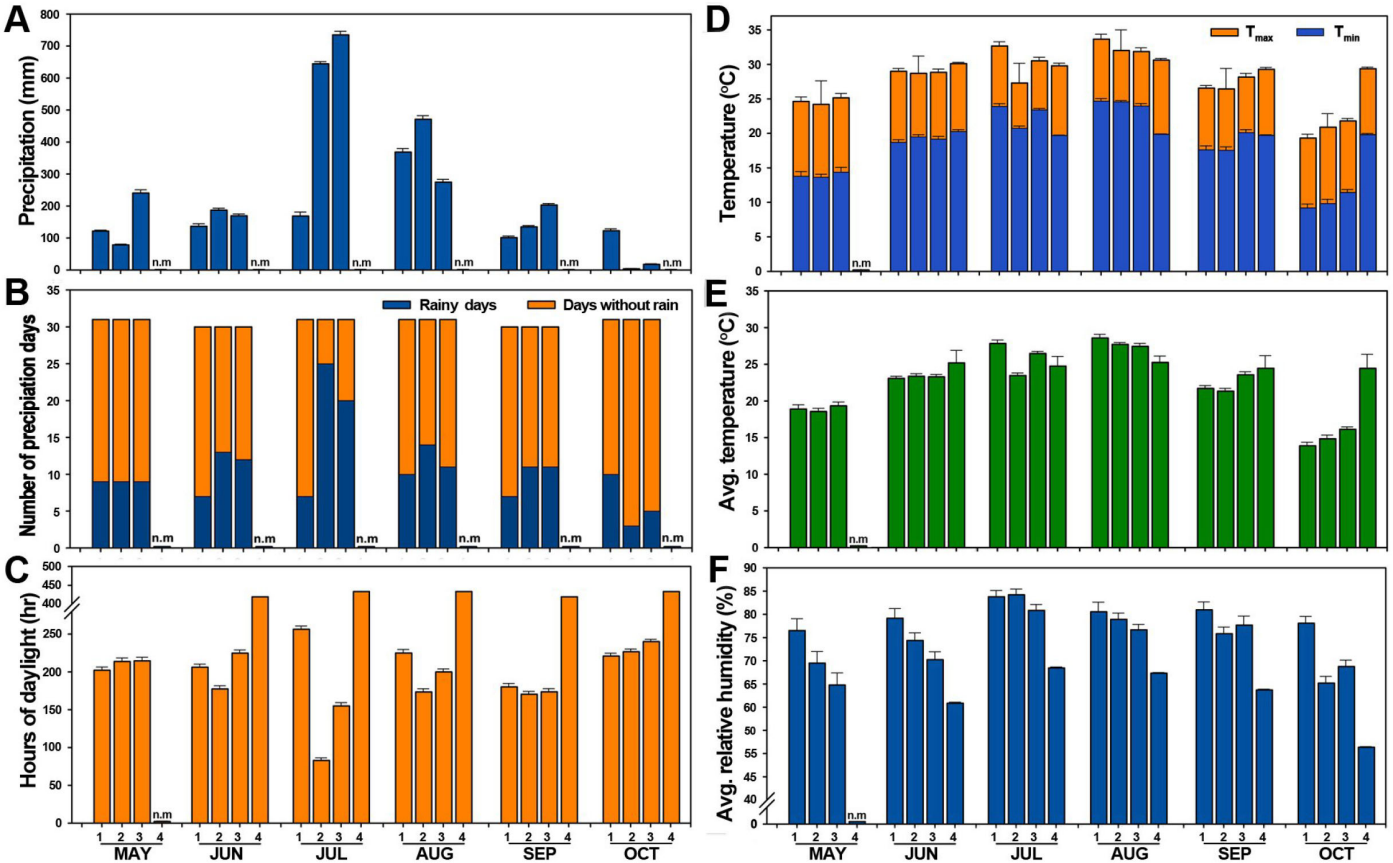
Graphical representation of monthly average changes in meteorological factors. Climate factors were measured from May to October during the 18-Pf, 20-Pf, 23-Pf, and 23-PMAG cultivation years. **(A)** Precipitation, **(B)** number of precipitation days, **(C)** hours of daylight, **(D)** high and minimum temperature, **(E)** average temperature, and **(F)** relative humidity. 1, paddy field in 2018; 2, paddy field in 2020; 3, paddy field in 2023; 4, plant phenotypic measuring automated greenhouse in 2023; *T*_max_, maximum temperature; *T*_min_, minimum temperature.

Among the analyzed meteorological information in **Supplementary Figure S4**, the most characteristic feature was Korea’s June and July precipitation data in 2018, showing less precipitation and number of precipitation days than the other years (20-Pf and 23-Pf), but a high temperature and humid dry rainy season environment was formed. Daily weather information was converted to monthly average conditions and analyzed to further investigate crop reproductive characteristics caused by climate factor fluctuations (**Figure 7**). It allowed us to understand the potential effects of predicted climate changes on reproductive growth characteristics around these averages. Analysis of monthly climate factors from May to October by cultivation year showed that the most striking feature was the change in precipitation in July (**Figure 7A**), measuring 169.1 mm in 2018, 644.7 mm in 2020, and 735 mm in 2023, respectively. Although precipitation was uneven across all months, precipitation in July 2018 was observed to be less in the range of 475.6 to 565.9 mm compared to other cultivation years, showing the greatest variability. The number of precipitation days in July (**Figure 7B**) was 7 in 2018, 25 in 2020, and 20 in 2023, representing 22.6%, 80.6%, and 64.5% of the total precipitation days in July, respectively.

Compared to other cultivation years (**Figure 7C**), the number of precipitation days in 2018 decreased by 13~18 days (42% to 58.1%). The hours of daylight in July 2018, when it rained less, was 256.6 h, representing increases of 308% and 165% compared to 83.1 h in 2020 and 155.1 h in 2023, respectively. In addition, in July, the highest temperature was 2.15°C to 5.34°C (**Figure 7D**), the lowest temperature was 0.5°C to 3.17°C, and the average temperature was 1.36°C to 4.37°C. Humidity did not show a significant difference from 81% to 84% by cultivation year. However, 2020 and 2023 showed more precipitation than the normal precipitation of 302.8 mm in July, and the hours of daylight was similar to the normal year in 2023 but less in 2020. The two analyzed years also showed differences from the normal climate (**Supplementary Table S7**). Summarizing the above climate information, the fluctuations in the number of days of precipitation and rain affected the hours of daylight and showed a significant correlation with temperature changes. In the 23-PMAG that controlled the rice growth environment (**Supplementary Figure S1**), the environment was kept constant within the range of set values such as temperature, humidity, and water supply, and environmental stress was minimized during the plant growth period. Consequently, the comparative assessment between the static-optimized 23-PMAG paradigm and the dynamic-stochastic field environments of 2018 and 2020 underscores that phenotypic manifestation in rice is not a uniform consequence of environmental fluctuations but a genotype-dependent recalibration of developmental trajectories. Rather than a generalized consequence of “climate change,” these results reveal that the reproductive success of each maturity group is dictated by a specific environmental–phenotypic covariance.

### 3.5 Correlation analysis between environmental drivers and reproductive traits

**Figure 8:**
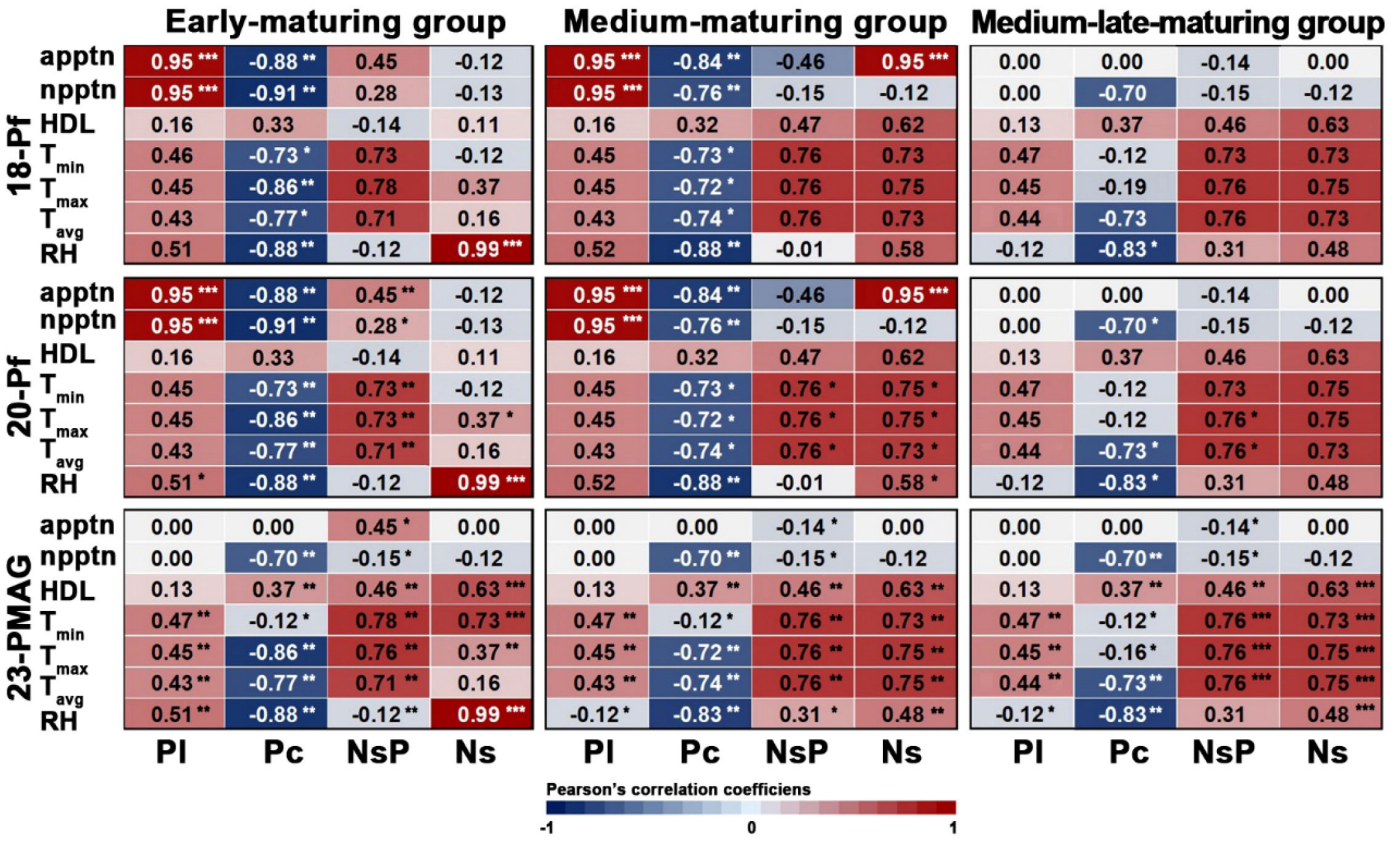
Pearson’s correlation analysis between environmental factors and rice reproductive traits across the three maturing groups and three environments. Within each heatmap, the *y*-axis lists seven environmental factors [precipitation (apptn), number of precipitation days (npptn), hours of daylight (HDL), min (*T*_min_), max (*T*_max_), and avg temperature (*T*_avg_), and relative humidity (RH)], and the *x*-axis lists four reproductive traits [panicle length (Pl), panicle count (Pc), seeds per panicle (NsP), and total seeds (Ns)]. The color of each cell indicates the Pearson correlation coefficient (*r*), with a diverging color scale from blue (strong negative correlation, −1.0) to red (strong positive correlation, +1.0), as shown in the color bar on the right. The numerical value of the correlation coefficient is displayed in each cell. Asterisks denote the statistical significance of the correlation: *p < 0.05, **p < 0.01, and ***p < 0.001. For the 23-PMAG environment, correlation coefficients for precipitation-related factors are 0.00 as precipitation was controlled and constant. 18-Pf, paddy field in 2018; 20-Pf, paddy field in 2020; 23-PMAG, plant phenotypic-measuring automated greenhouse in 2023.

To elucidate the mechanistic links between climatic fluctuations and reproductive performance, a Pearson correlation heatmap was constructed (**Figure 8**). The analysis revealed that the early- (E) and medium (M)-maturing groups were highly sensitive to precipitation patterns (apptn and npptn) under field conditions (18-Pf and 20-Pf). Specifically, both E and M groups exhibited a robust positive correlation (*r* = 0.95, *p* < 0.001) between apptn/npptn and Pl, alongside a significant negative correlation (*r* = −0.91, *p* < 0.01) with Pc. This suggests a developmental trade-off where increased rainfall promotes panicle elongation at the expense of tiller-derived panicle numbers in these cohorts.

In stark contrast, the ML group exhibited a near-zero correlation (*r* = 0.00) with precipitation variables under field conditions, indicating a superior environmental buffering capacity or a distinct ontogenetic threshold compared to the E and M groups. Furthermore, RH emerged as a critical driver for total seed number (Ns) in the early group, showing an exceptionally high positive correlation (*r* = 0.99, *p* < 0.001) across all environments. Under the homeostatic CEA paradigm (23-PMAG), the influence of stochastic precipitation was eliminated, shifting the primary drivers to HDL and temperature (*T*_min_, *T*_max_, *T*_avg_). Across all maturity groups in the CEA system, HDL was significantly and positively correlated with Pc (*r* = 0.37, *p* < 0.01) and Ns (*r* = 0.63, *p* < 0.001), confirming that optimized light interception is a fundamental prerequisite for maximizing reproductive output in controlled environments.

## 4 Discussion

Worldwide, climate change is causing severe damage to rice production by increasing the frequency and intensity of natural disasters (Myeong, 2018). While the multifaceted impacts of climate change cannot be artificially prevented, a key countermeasure is the development of varieties with excellent phenotypic plasticity to adapt and survive in changing environments (Li et al., 2019; Wang et al., 2021). Recently, the climate in Korea has been transitioning toward a hot and humid subtropical climate, characterized by irregular short-term heavy rainfall and typhoons during the rice growing stage (Weather Spark, https://weatherspark.com/countries/KR; Hong et al., 2023). This climatic shift is a direct cause of reduced yields by inducing heat stress or flood damage, particularly during the rice plant’s pollination and grain-filling stages. Therefore, this study evaluated 100 commercial temperate Japonica rice cultivars from three maturing groups. We aimed to analyze and visualize their phenotypic plasticity in response to environmental changes and, through climate change analysis, to identify genotypes with superior phenotypic plasticity according to changes in the cultivation environment (Laitinen, 2024).

In interpreting the results of this study, it is imperative to shift from viewing the experimental design as a simple “experimental-control” relationship to approaching it as a comparative study of two fundamentally different environmental paradigms. The absence of a 2023 field control is a clear limitation from a traditional perspective. However, the core of this study is that the two paradigms themselves—the “dynamic-stochastic field environment” and the “static-optimized controlled environment agriculture (CEA) environment”—are the independent subjects of comparison. The field environment, with its diurnal temperature fluctuations, varying light conditions, and unpredictable precipitation, as well as complex stressors like soil microbial communities, pest pressure, and interplant competition, acts as an evolutionary pressure that fosters the development of phenotypic plasticity and stress memory in plants. In contrast, the CEA environment seeks to artificially eliminate this variability to maintain homeostasis. However, this perfect constancy can imply the absence of key external cues, or “zeitgebers,” that entrain the plant’s endogenous circadian clock (Harmer, 2009; Suzuki et al., 2014; Creux and Harmer, 2019). This can lead to a paradoxical stress known as “the paradox of perfection,” which, despite optimal conditions, may result in metabolic imbalances and growth inhibition. From this viewpoint, the attenuated genotype × environment (G × E) interaction signal observed in this study is not an indication of “no G × E,” but is in itself a significant finding. It can be interpreted as evidence that the static CEA environment acted as a powerful filter, suppressing the expression of the genetic potential for plasticity that would otherwise be manifested under diverse conditions. In other words, as the environmental variance (“E”) in the G × E equation becomes a near-constant, the opportunity for differential genotypic (“G”) responses to be expressed is fundamentally suppressed. Therefore, the phenotypic differences observed between the field and greenhouse are not merely the effect of a single variable like water management but are the result of a complex, holistic physiological response of the same genotypes to two fundamentally different “survival philosophies”: “variability” versus “constancy.” This implies that breeding future CEA-specific cultivars must consider not just yield but also the “hidden” aspects of environmental responsiveness and adaptability.

To investigate the performance of Korean rice cultivars under contrasting agricultural paradigms, we conducted a comparative assessment of reproductive traits across the “dynamic-stochastic field environment” (18-Pf and 20-Pf) and the “static-optimized controlled environment agriculture (CEA)” system (23-PMAG). Across the 100 genotypes, the transition to the controlled greenhouse environment resulted in a significant increase in Pc, with an overall average increase of 36.4% compared to the field average. When stratified by maturity group, Pc augmented by 39.0% in the early-maturing group (field mean 11.35 to PMAG 15.78), 37.2% in the medium-maturing group (field mean 11.58 to PMAG 15.89), and 32.9% in the ML group (field mean 11.87 to PMAG 15.78). This pronounced surge in Pc is interpreted as a direct physiological consequence of facilitated tiller formation, bolstered by the stable nutrient supply and optimized water management inherent to the CEA system. Conversely, the total Ns manifested a highly differentiated response closely coupled with the developmental timing and genetic background of each maturity group. While the early-maturing group exhibited a robust 43.1% increase in Ns (field mean 1,208.96 to PMAG 1,729.84), indicating high responsiveness to environment optimization, the medium-maturing group showed a more moderate increase of 27.2%6. Notably, the ML group showed a statistically marginal variation of only 3.2% (field mean 1,245.99 to PMAG 1,285.45). This indicates that in genotypes with longer growth durations, the gain in Pc was effectively offset by a concurrent reduction in Pl and the number of filled spikelets per panicle (NsP) under controlled conditions. This phenomenon, often termed the “paradox of perfection,” suggests that the absence of specific environmental cues or “zeitgebers”—such as the diurnal temperature fluctuations typical of field conditions—may hinder the physiological transition required for late-stage reproductive development, including panicle elongation and spikelet differentiation. Such phenotypic variations resulting from G × E interaction underscore the genotypic differences in plasticity and emphasize the necessity of considering environmental responsiveness when breeding future CEA-specific cultivars (Kusmec et al., 2018; Arnold et al., 2019; Huang et al., 2021).

The differential manifestation of reproductive plasticity observed across the three cohorts underscores a fundamental divergence in the adaptive landscapes of Korean Japonica rice cultivars. While early-maturing genotypes displayed high environmental sensitivity—facilitating a rapid yield surge (43.1% increase in Ns) upon the removal of abiotic stressors in the CEA system—the ML genotypes remained relatively unresponsive. This phenomenon suggests that ML cultivars possess a genetic architecture that is highly canalized to withstand the stochastic fluctuations of field environments, such as the irregular rainfall and thermal shifts observed in 2018 and 2020 (Hallgrimsson et al., 2019; Anholt and Mackay, 2023). In the absence of specific environmental zeitgebers (e.g., diurnal temperature oscillations and spectral variability) within the PMAG system, these ML genotypes appear to experience a physiological mismatch, which we term the “paradox of perfection.” This lack of response is not merely “stability” but likely reflects an ontogenetic constraint where the genetic potential for panicle elongation (Pl) and spikelet differentiation (NsP) is suppressed by the lack of external cues required to trigger late-stage reproductive transitions. Consequently, our findings demonstrate that the G × E interaction signal is driven by inherent differences in plasticity syndromes tied to maturity-related alleles, highlighting the necessity for breeding programs to select for “environmentally entrained” cultivars specifically for high-precision agricultural paradigms.

When comparing the direction of change for each cultivar relative to the mean, we identified movement from left to right or vice versa, with infrequent cases where they remained in the same direction. Values to the left of the mean represent a decreasing pattern, while values to the right represent an increasing pattern. The length of the line connecting these points for a given cultivar varied from short to long. This difference in length can be explained by the magnitude of phenotypic variation of the cultivar; a long length suggests high variability depending on the cultivation conditions. These results show that each maturing group has specific phenotypic variation by cultivar and a complex structure. Simplifying the results shown in **Figure 5A**, a comparison of the variance of phenotypic variation shows that the variance according to the cultivation year of Pf and greenhouse cultivation is different. Unlike the medium–late-maturing group, which has constant variance and size (except in 23-PMAG), the early-maturing and medium-maturing groups have different distributions and sizes. This suggests that the degree and pattern of response to stress environments differ genetically among the maturing groups. In addition, the total distance of the positional change also has different variance for each of the three maturing groups (excluding extreme locations), which means that they have specific phenotypic plasticity due to G × E depending on the cultivation environment (Kusmec et al., 2018; Huang et al., 2021; Hildebrandt, 2023).

The phenotypic variation of reproductive traits showed a specific correlation with changes in climate factors such as precipitation, number of rainy days, daylight hours, temperature, and relative humidity during the cultivation years. Climatic differences in July were particularly noticeable. July 2020 was similar to a general rainy season but with a longer duration (25 rainy days) and higher precipitation from local heavy rainfall and typhoons (set as environmental condition 1, 20-Pf). In contrast, July 2018 showed a high temperature and high humidity climate with less precipitation and fewer rainy days than the general rainy season, resulting in an increase in daylight hours (set as environmental condition 2, 18-Pf). The automated greenhouse for rice phenotyping was set as the optimal environmental condition for rice growth (set as environmental condition 3, 23-PMAG). The responses of the cultivars to these different environments resulted in complex phenotypic variations. This extends beyond simple changes in mean values to how the distribution of variation within the population itself was altered. For instance, if a bimodal distribution with two peaks appeared under a specific environment (e.g., the hot and dry conditions of 18-Pf), it could suggest that the population’s response to that stress split into “sensitive” and “resistant” groups. Conversely, a narrowing of the distribution would imply that the environment exerted stabilizing selection, favoring only individuals with a specific phenotype.

The formal correlation analysis provided in **Figure 8** facilitates a robust predictive framework for rice productivity under shifting climatic regimes. As the East Asian summer monsoon (Changma) is projected to exhibit increased intensity and extended duration due to anthropogenic climate change, understanding the environmental sensitivity of reproductive traits is paramount. Our findings reveal a distinct phenotypic buffering capacity in the ML group, which exhibited near-zero correlation (*r* ≈ 0.00) between precipitation parameters (apptn, npptn) and reproductive output (**Figure 8**). This suggests that ML cultivars possess superior genetic robustness or ontogenetic resilience, making them ideal candidates for maintaining yield stability in regions facing increasingly volatile rainfall patterns. Conversely, the early- and medium-maturing groups displayed a significant developmental trade-off. while increased precipitation was positively correlated with panicle length (Pl, *r* = 0.95), it simultaneously triggered a severe reduction in panicle count (Pc, *r* = −0.91) (**Figure 8**). This negative environmental–phenotypic covariance indicates that for these cohorts, a prolonged monsoon season could lead to a “sink-limited” architecture, where the gain in individual panicle size fails to compensate for the loss in total productive tillers. Furthermore, the exceptionally high correlation between RH and Ns in early cultivars (*r* = 0.99) underscores their vulnerability to the humid conditions often associated with late-season typhoons. In the context of CEA, the significant positive correlation between HDL and reproductive traits (Pc, Ns) underscores that the “paradox of perfection” can be mitigated by optimizing spectral interception. These quantitative insights suggest that future breeding programs should prioritize the incorporation of “plasticity alleles” from ML genotypes into early-maturing backgrounds to enhance climate-resilient architecture. Consequently, our multi-environmental framework serves as a vital tool for the phenotypic prediction and targeted selection of rice varieties capable of traversing the complex fitness landscapes of future agroecosystems.

Agricultural productivity is intrinsically constrained by the dynamic nature of the cultivation environment (Kopeć, 2024). While anthropogenic interventions can partially modulate microclimatic conditions, crops must possess the innate capacity to adapt to fluctuating and novel environmental regimes (Li et al., 2019; Wang et al., 2021; Hang et al., 2024). The phenotype serves as the integrated manifestation of genotype-by-environment (G × E) interactions, representing the physiological and morphological expressions of genomic regulatory networks responding to exogenous stimuli (Shinozaki and Yamaguchi-Shinozaki, 2022). Consequently, elucidating the intricate interplay between genotype and environment is fundamental to assessing adaptive fitness and survival strategies. Through these adaptive mechanisms, crops maintain reproductive success under climatic shifts, ensuring the transmission of favorable alleles to subsequent generations (Munir et al., 2025). Directional selection pressure within a given environment favors individuals with superior fitness, thereby reshaping the population’s gene pool (Brendel et al., 2021). In this context, phenotypic variation within a taxon reflects its plastic potential, which is instrumental in mitigating the adverse effects of anthropogenic climate change (Laitinen, 2024). When selective pressures are exerted on these variations, the population exhibits evolutionary resilience through phenological and structural modifications (González Guzmán et al., 2022).

Enhanced phenotypic variance is positively correlated with the probability of survival under environmental perturbations (Zhang et al., 2024). However, modern elite cultivars, specifically bred for high-input and high-yield stability in optimized environments, often exhibit attenuated responses to rapid environmental fluctuations (Begna, 2021; Huang et al., 2021; Hang et al., 2024). This phenomenon is largely attributed to the genetic bottlenecks and subsequent genetic erosion incurred since the “Green Revolution”; intensive selection for yield-related traits in benign environments has inadvertently purged alleles essential for abiotic and biotic stress resilience. Such vulnerabilities include diminished nutrient-use efficiency, compromised innate immunity, and hypersensitivity to thermal shifts, all of which impede transgenerational fitness (Begna, 2021). From an evolutionary perspective, although a specific trait may offer higher theoretical fitness, its fixation may be hindered if the transition requires traversing “valleys” of maladaptive intermediate phenotypes within the fitness landscape (Arnold et al., 2019; Wang et al., 2021). Therefore, broadening the phenotypic and genetic base is imperative for sustaining crop productivity. Recent genomic perspectives suggest that “re-wilding” crop genomes—incorporating ancestral plastic alleles—may be a prerequisite for restoring the adaptive mechanisms lost during domestication (Mansoor and Chung, 2024). Furthermore, navigating complex fitness landscapes under stochastic climate regimes necessitates a profound understanding of how phenotypic plasticity can bridge adaptive gaps to reach new fitness optima (Wang et al., 2021; Mansoor et al., 2025).

Crops must adapt to climate change and find conditions that allow them to survive. Climate changes and increased phenotypic variations can improve the viability of crops. By expressing unique phenotypic plasticity, “plasticity populations” can adapt to new conditions and promote evolutionary adaptation. Increasing these phenotypic variations can significantly aid in the development of resilient varieties for future climates by distinguishing between flexible and less flexible varieties in response to climate change, thereby understanding the phenotypic plasticity of crops in relation to environmental changes.

## 5 Conclusions

This study established a novel, multi-environmental analytical framework by rigorously assessing 100 Korean rice genotypes across a precision-controlled CEA system (23-PMAG) and stochastic field environments (18/20-Pf) to elucidate the quantitative mechanisms of reproductive plasticity. Moving beyond the reductionist view that climatic shifts simply induce phenotypic variation, these results provide robust evidence for a genotype-specific recalibration of developmental trajectories dictated by the genetic architecture of distinct maturity groups. Utilizing KDE and variability analysis, we found that rainfall-related climatic factors—affecting precipitation days, temperature, and daylight hours—significantly recalibrated phenotypic expression. Compared to the homeostatic 23-PMAG environment, both dry (2018) and heavy rainfall (2020) conditions resulted in lateral migration of KDE peaks and the emergence of bimodal distributions, indicating clear interspecies differentiation in response to environmental fluctuations.

Quantitative insights derived from Pearson’s correlation heatmap reveal that early- (E) and medium (M)-maturing groups exhibit an acute sensitivity to precipitation patterns (apptn, npptn), manifesting as a pronounced developmental trade-off between panicle elongation (*r* = 0.95) and tiller-derived panicle count (*r* = −0.91). In contrast, the ML group demonstrated superior environmental buffering capacity, with correlation coefficients converging toward zero for the same field variables, thereby signifying inherent genetic robustness. Contrary to the conventional expectation that optimized CEA environments maximize yield potential for all genotypes, the ML group experienced suppressed reproductive development compared to field conditions due to the absence of specific environmental zeitgebers. This rigorously validates that the phenomenon of genetic canalization must be accounted for when breeding future cultivars tailored for precision agricultural systems. The phenotypic plasticity models established herein provide a robust predictive framework for determining the developmental strategies each maturity group will adopt under shifting climate scenarios, such as intensified rainfall and diminished solar radiation. Specifically, a strategic breeding paradigm that integrates the opportunistic plasticity of early genotypes with the stability alleles of ML genotypes will be instrumental for developing next-generation climate-resilient cultivars. Ultimately, this research notably demonstrates the utility of high-resolution automated phenotyping and substantiates that its integration with multi-environmental datasets is indispensable for decoding complex environmental–phenotypic covariance. The data-driven plasticity roadmap presented here serves as a critical academic foundation for ensuring the stability of rice production in future agroecosystems.

## Data Availability

The original contributions presented in the study are included in the article/**Supplementary Material**. Further inquiries can be directed to the corresponding authors.
