# Supplementary material for: Evaluating phenotypic plasticity of reproductive traits among Korean rice cultivars under diverse climatic conditions
Source: Front Plant Sci. 2026 Mar 19;17:1697493. doi: 10.3389/fpls.2026.1697493 (PMC13044014; doi:10.3389/fpls.2026.1697493)
Supplement: Supplementary file 1 [file DataSheet1.pdf]

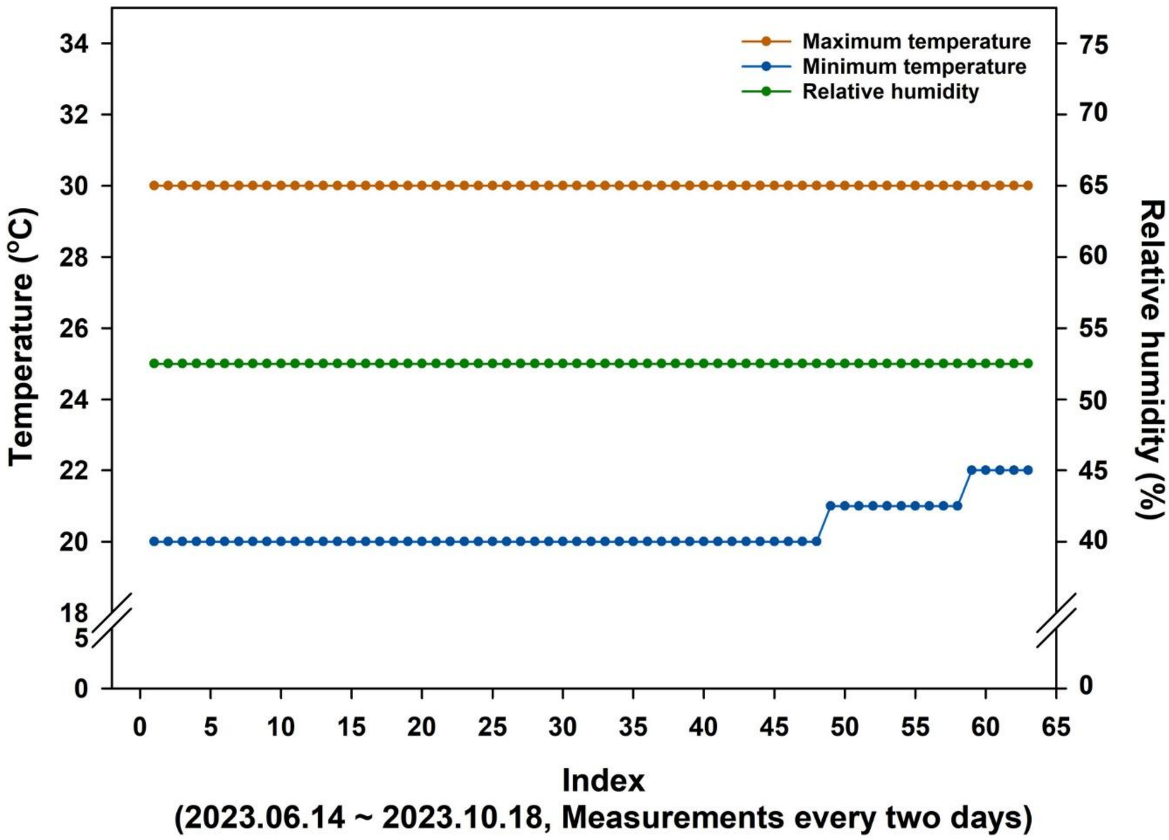

Fig. S1. Environment settings of the plant phenotypic measuring automated greenhouse (PMAG) during the rice growth period. Temperature and humidity measurements were taken from 14th June to 18th October 18, 2023.
