# Supplementary material for: Evaluating phenotypic plasticity of reproductive traits among Korean rice cultivars under diverse climatic conditions
Source: Front Plant Sci. 2026 Mar 19;17:1697493. doi: 10.3389/fpls.2026.1697493 (PMC13044014; doi:10.3389/fpls.2026.1697493)
Supplement: Supplementary file 2 [file DataSheet2.pdf]

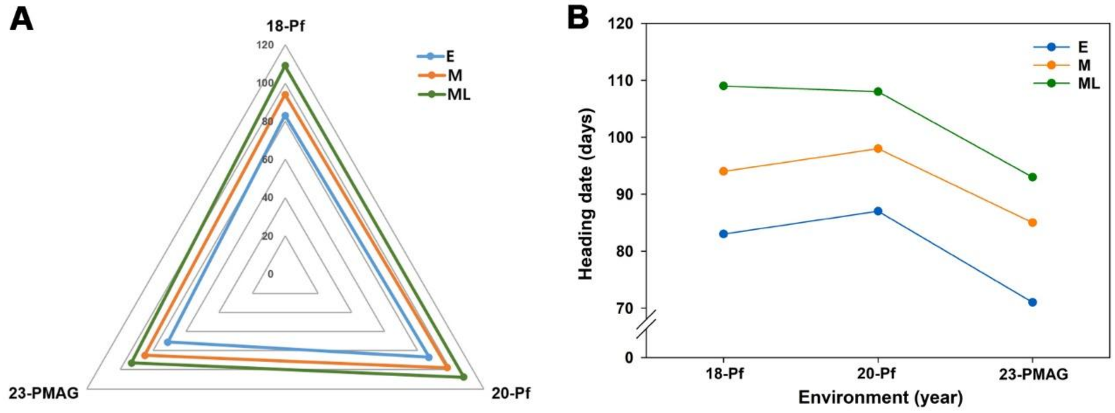

Fig. S2. Relationship between three maturing groups and cultivation years in heading dates. (A) Radar chart of the average heading dates in three maturing groups with different cultivation years. (B) Graphical representation of both the three maturing groups and average heading dates in response to changing environments (different cultivation years). E, early-maturing group; M, medium-maturing group; ML, medium-late-maturing group; 18-Pf, paddy field in 2018; 20-Pf, paddy field in 2020; 23-PMAG, plant phenotypic-measuring automated greenhouse in 2023.
