# Supplementary material for: Evaluating phenotypic plasticity of reproductive traits among Korean rice cultivars under diverse climatic conditions
Source: Front Plant Sci. 2026 Mar 19;17:1697493. doi: 10.3389/fpls.2026.1697493 (PMC13044014; doi:10.3389/fpls.2026.1697493)
Supplement: Supplementary file 3 [file DataSheet3.pdf]

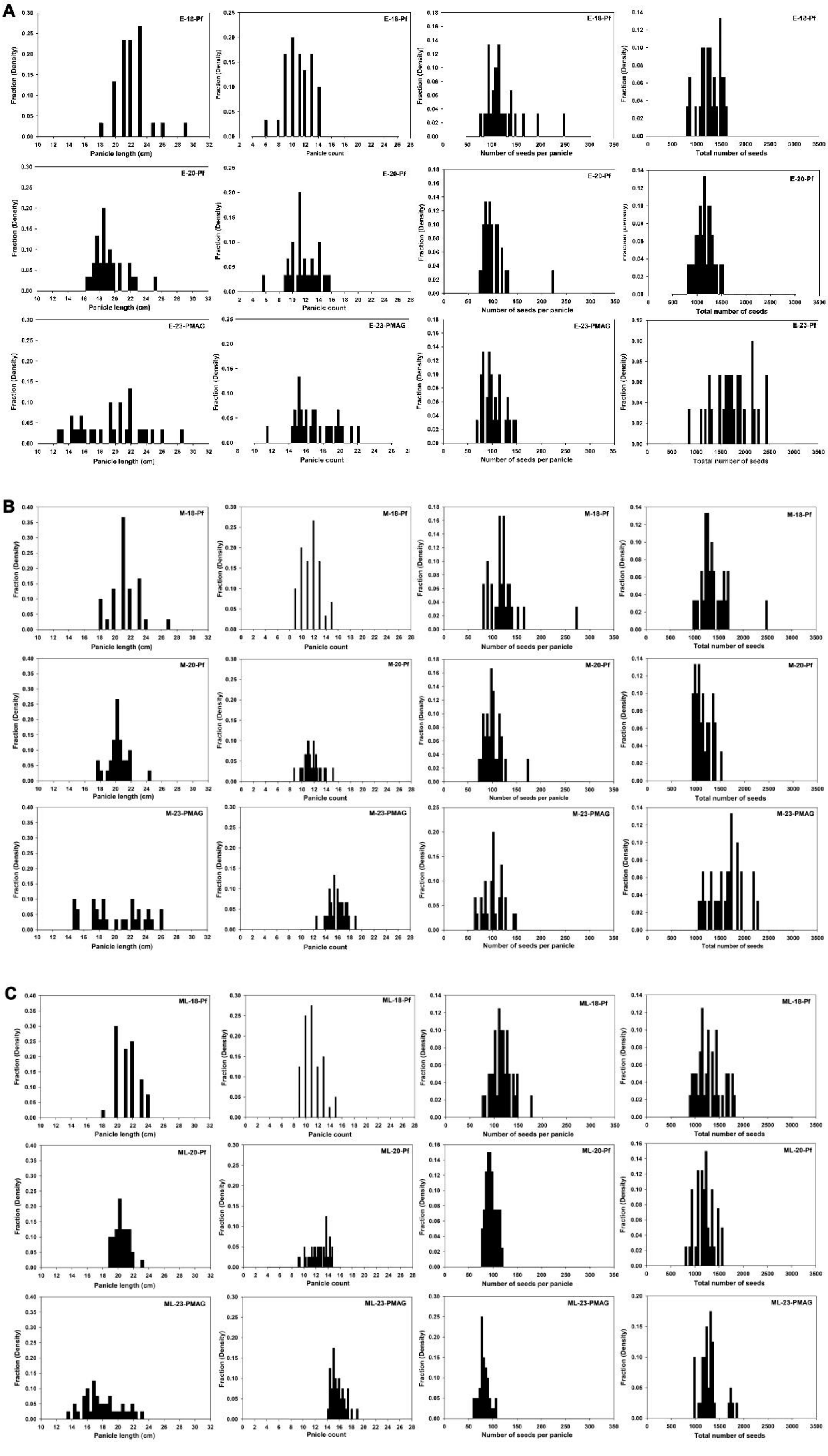

Fig. S3. Histogram displaying the variation of reproductive four traits in three maturing groups with different cultivation years. (A) Early-maturing group, (B) medium-maturing group, (C) medium-late-maturing group. 18-Pf, paddy field in 2018; 20-Pf, paddy field in 2020; 23-PMAG, plant phenotypic-measuring automated greenhouse in 2013.
