# Supplementary material for: Evaluating phenotypic plasticity of reproductive traits among Korean rice cultivars under diverse climatic conditions
Source: Front Plant Sci. 2026 Mar 19;17:1697493. doi: 10.3389/fpls.2026.1697493 (PMC13044014; doi:10.3389/fpls.2026.1697493)
Supplement: Supplementary file 4 [file DataSheet4.pdf]

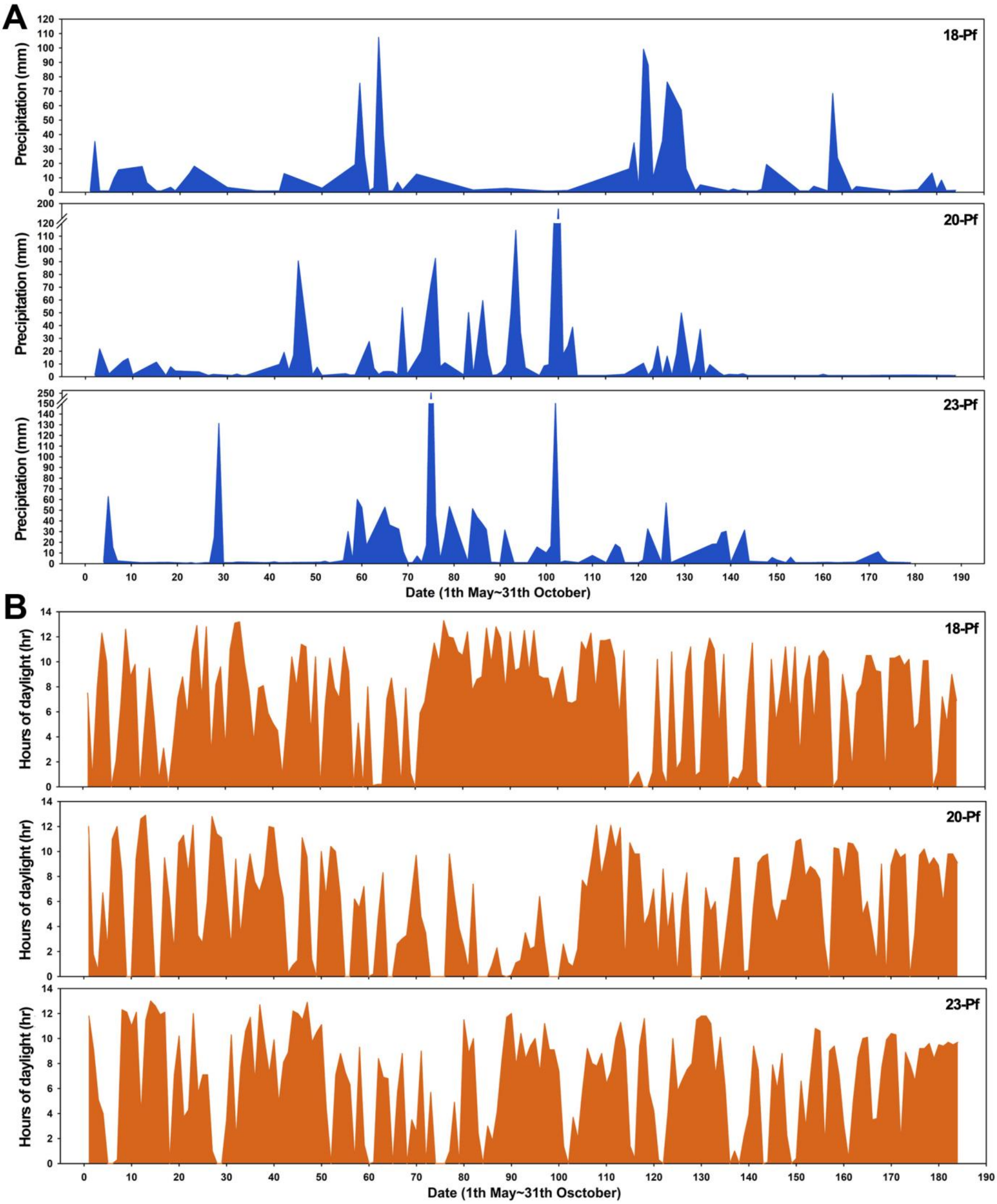

A
18-Pf
20-Pf
23-Pf
Precipitation (mm)
Date (1th May~31th October)
B
18-Pf
20-Pf
23-Pf
Hours of daylight (hr)
Date (1th May~31th Osctober)

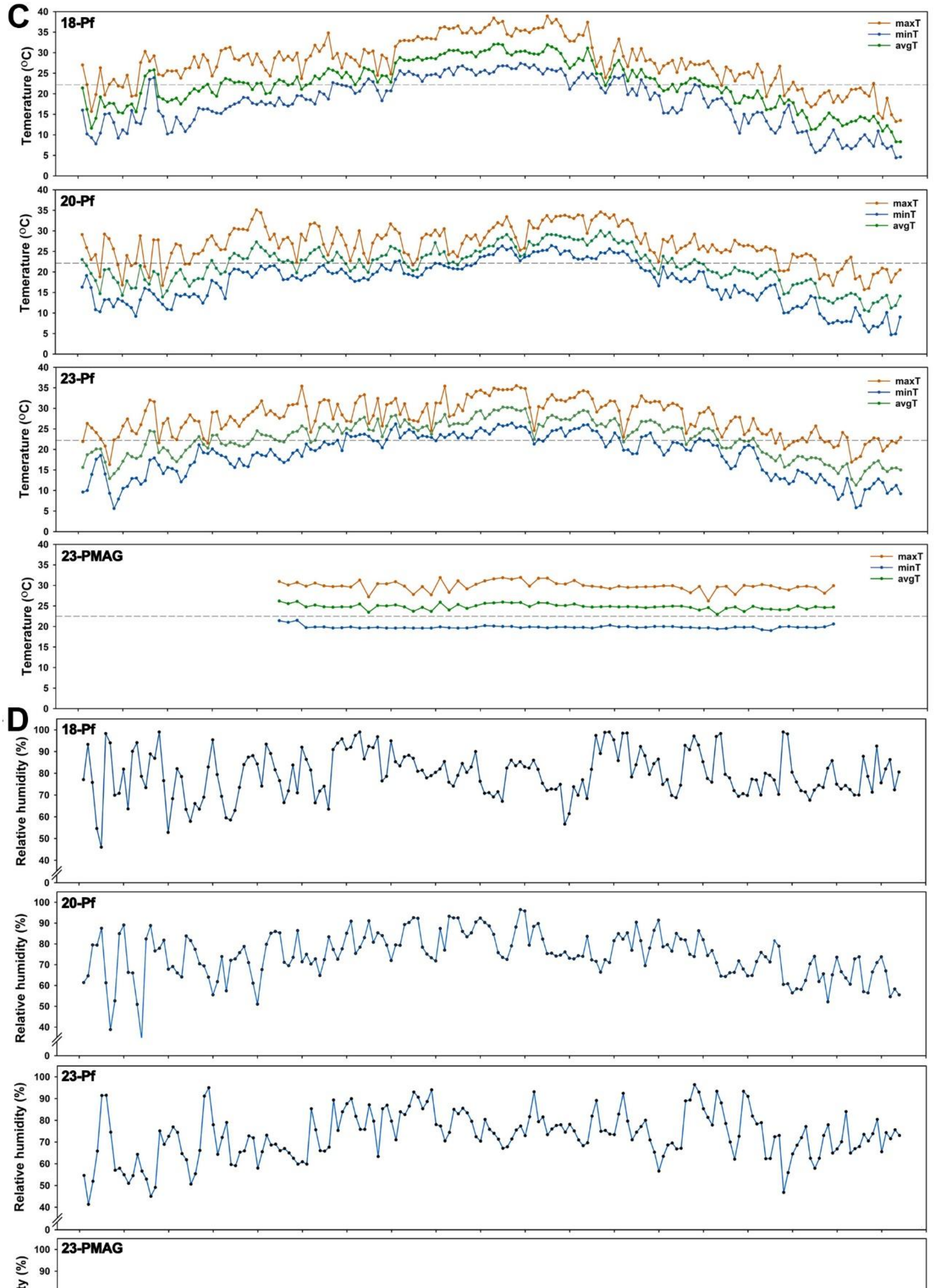
C
18-Pf
20-Pf
23-Pf
23-PMAG
maxT
minT
avgT
Temerature (OC)
D
Relative humidity (%)
Date (1th May~31th October)

Fig. S4. Graphical representation of daily changes in meteorological factors. Climate factors were measured from May to October during 18-Pf, 20-Pf, 23-Pf, and 23-PMAG cultivation years. (A) Precipitation, (B) number of precipitation days, (C) high and minimum temperature, (D) average relative humidity. 18-Pf, paddy field in 2018; 20-Pf, paddy field in 2020; 23-Pf, paddy-field in 2023; 23-PMAG, plant phenotypic measuring automated greenhouse in 2023; avgT average daily temperature; (continued) maxT, daily maximum temperature, minT, daily minimum temperature.
